# An Unusual Presentation of Catatonia-Like Behavior: Differentiating Malingering from Catatonia

**DOI:** 10.1155/2021/1860757

**Published:** 2021-11-29

**Authors:** Andy Y. Wang, Urrooj H. Rehman

**Affiliations:** Department of Psychiatry, BayRidge Hospital, Beth Israel Lahey Health, Lynn, MA, USA

## Abstract

Malingering involves the intentional production of physical or psychological behaviors due to motivation from external incentives, posing unique challenges to healthcare. Although malingering as an entity has been well studied, the current literature does not explore the intentional production of catatonia-like behavior or how to differentiate malingering from catatonia. Here, we describe a 45-year-old female who was admitted to an acute psychiatric hospital with a complex presentation of catatonia-like signs that was ultimately thought to be volitional behavior, resulting in a diagnosis of malingering. We highlight the important factors considered in her presentation, the differences between her behaviors and true catatonia, and other important differential diagnoses to consider. Although a diagnosis of malingering is difficult to make, we underscore the importance of reaching this conclusion in order to avoid unnecessary and potentially harmful medical interventions. We stress the importance of shifting focus from medical management to more appropriate patient goals such as providing social services and treatment of other underlying psychiatric illnesses.

## 1. Introduction

Malingering, the feigning of psychiatric illness for external gain or avoidance of punishment, has been long recognized in human history and popularized in classical literature. Shakespeare's Hamlet disguised himself under fits of madness, and Odysseus avoided fighting in the Trojan war by pretending to be mentally ill. In psychiatric studies, malingering has been well described. However, there is a paucity of scientific literature regarding the feigning of catatonia-like symptoms, potentially because catatonic symptoms are less well understood by the public and more difficult to maintain for long periods of time. Catatonia was first described by Karl Kahlbaum in 1874 as a complex and diverse syndrome of motor disturbance with an inability to move normally despite full ability, in an underlying psychiatric or medical disorder [1]. Treatment involves resolving the underlying psychiatric or medical issue, benzodiazepines, and electroconvulsive therapy [2]. Currently, there is little guidance on how to differentiate malingering with catatonia-like behavior from catatonia.

Here, we present a case report of a patient who presented with catatonia-like behaviors that were inconsistent, diverse, and most likely to be volitional. We detail the important features of her presentation, highlight differences between her presentation and catatonia, other differential diagnoses to consider, and the importance of recognizing malingering in such patients to avoid unnecessary treatments and to shift the focus of care to more appropriate patient goals.

## 2. Case Presentation

The patient was a 45-year-old female who was admitted to an acute psychiatric hospital with depressed mood, suicidal ideation, and auditory hallucinations. She was discharged five days prior from a different hospital, and according to previous records, has been repeatedly hospitalized within the last 12 months totaling 18 emergency department visits. Her psychiatric history was significant for benzodiazepine use disorder, opiate use disorder, borderline personality disorder, and major depressive disorder. Her medications on admission included clonazepam, clonidine, buprenorphine and naloxone, fluoxetine, gabapentin, and trazodone. Her social history included unemployment and homelessness without family support.

During the admission, the patient exhibited a wide range of odd behaviors that will be described in the categories of catatonia-like signs, psychotic signs, and seizure-like signs. Catatonia-like signs included (1) a flat affect with psychomotor retardation and fluctuating mutism, (2) complete lack of social contact with other patients for the entirety of the hospital stay, (3) twitching and eye grimacing with shoulder shrugging, (4) posturing and immobility, and (5) diaphoresis. These severe signs persisted throughout her hospital stay and were so convincing of catatonia that multiple attendings, nurses, and staff held special meetings to specifically review her catatonic status. Additional consultants were recruited to discuss her case at length. Furthermore, these catatonic symptoms resulted in the patient's hospital stay lasting almost two months as the appropriate treatment and disposition were considered. Psychotic signs included (1) mouthing words and seemingly responding to internal stimuli, (2) thought blocking, and (3) reported auditory hallucinations. Seizure-like behavior included episodes of jerking head back and forth with her eyes fluttering without tongue biting. She was conversant during the episodes, and when seen by a consultant, her exam was benign.

The signs described above were complicated by subtle clues pointing towards volitional behavior, as confirmed by multiple staff members and psychiatry attendings involved in her care. Her external goals appeared to be to gain access to benzodiazepines as well as to shelter and food. Evidence for this included repeatedly verbalizing that she was withdrawing and needed clonazepam, and selectively choosing only the benzodiazepines, clonidine, and gabapentin from the cup of medications given to her every day while refusing the other medications. Discussion of her case with a relative revealed that she had been overusing her prescription clonazepam and opioids. Throughout her hospital stay, she ate every meal without prompting or interruption from her other behaviors, unusual of catatonia. Furthermore, she was not eager for discharge and appeared to prolong the hospital stay willingly. In terms of her catatonia-like behaviors, the patient would lapse into psychomotor retardation, mutism, and repetitive movements such as grimacing and eye twitching during uncomfortable or uninteresting topics of conversation. She would then lapse out of these actions abruptly and speak lucidly about urgent issues she wanted to vocalize. This was similar to her psychotic symptoms, such as abruptly lapsing in and out of mumbling, mouthing words, and seemingly responding to internal stimuli. The use of risperidone did not seem to alleviate these psychotic behaviors. As for inconsistencies in her seizure-like activity, the patient would often have dramatic seizure events and then suddenly stand up after her seizure episodes and walk independently back to her room to position herself for an expected lorazepam injection. There was one incidence where staff members saw her purposefully pour water onto her pants to feign urinary incontinence during a seizure episode. In some of her more minor automatisms, she would shake and jerk one arm while the other arm would steadily hold onto food and drink without spillage.

The patient's case was discussed at length with all the attending psychiatrists, case managers, and hospital staff who were involved in her care. A catatonia specialist in the region was also consulted, who confirmed that she was likely to not have catatonia. Repeated administration of lorazepam as challenge tests seemed to have a limited effect on her catatonic and other behaviors and would instead seem to reinforce them. After a protracted hospital stay of two months, the patient was discharged to a shelter with proper social supports and psychiatric follow-up. Two and three months after discharge, the patient represented to two different hospitals at different cities for unusual medical concerns and requests for medications, leaving against medical advice each time, once walking out with a peripheral IV still in hand.

## 3. Discussion

The most important aspect of this case is the differentiation between malingering and catatonia. Principal features of catatonia include mutism, stupor, negativism, posturing, waxy flexibility, stereotypy, automatic obedience, ambitendency, echophenomena, and odd mannerisms [1]. The Diagnostic and Statistical Manual, 5th Edition (DSM-5) criteria are used for a diagnosis of catatonia, which includes greater than three of these clinical features [3]. The most sensitive and validated rating scale for catatonia is the Bush-Francis Catatonia Rating Scale [2]. The patient's Bush-Francis Catatonia Rating Scale wavered each day from 5 to 7, sometimes positive for immobility, grimacing, mutism, posturing, and diaphoresis, yet not in the typical presenting manner of patients with catatonia. As described earlier, malingering involves intentional production of false or grossly exaggerated physical or psychological problems that are motivated by external incentives [3]. The main clues in this case that pointed towards malingering included the presence of known motivating external incentives (benzodiazepines in the context of her substance use disorder, housing, and food), the fluctuating and circumstantial changes to her behavior, and inconsistency with any known psychiatric disorder. Furthermore, she was unresponsive to lorazepam challenge tests. Most importantly, these observations were made and confirmed by multiple staff members, case managers, and attending psychiatrists and discussed at length in team meetings.

Other differential diagnoses considered included major depressive disorder (MDD) with catatonia, schizophreniform disorder with catatonia, conversion disorder, and factitious disorder. Although the patient was previously diagnosed with MDD, psychotic features and catatonia-like behaviors were new in her medical record, less likely to be part of her long-standing MDD diagnosis, and inconsistent with her volitional behavior. Schizophreniform disorder was considered given her presentation of new positive and negative psychotic symptoms but is unusual to have an onset at her age, and her symptoms were unlike typical presentations. Conversion disorder with catatonia has been described before in the literature, but the patient had no acute stressor, and appeared to be volitional in her behavior. Factitious disorder imposed on self could be considered especially in consideration of her repeated hospitalizations, but the patient did not seem interested in assuming the sick role, consistently showing displeasure with the care team if her demands were not met. As opposed to factitious disorder in which symptoms are feigned to assume the sick role, patients who malinger seek to avoid difficult situations or gain external rewards [4]. Ultimately, the patient was diagnosed with malingering complicated by her existing psychiatric illnesses. Most of her behavior was thought to be purposeful and in service of acquiring benzodiazepines, housing, and food.

To the best of our knowledge, only one other case report in the literature has reported a patient who appeared to have feigned catatonia-like behavior in what was thought to be factitious disorder [5]. That patient presented with mutism, stupor, negativism, and withdrawn behavior, and staff noticed that she had episodes of spontaneous purposeful movement when not under direct supervision. Other case studies have discussed catatonia in association with conversion disorder, but not with purposeful feigning of catatonia [6–8]. Catatonia is difficult to malinger, and extended observation during hospitalization will usually reveal the patient to have inconsistencies in their behaviors [9].

Malingering is a difficult diagnosis for physicians to make given the clinical uncertainty of an unusual presentation, potential harm to the patient with a wrong diagnosis, fear of malpractice, and damage to the trust of the patient-doctor relationship. However, continued medical treatment for feigned symptoms also has its risks. Benzodiazepines were administered to our patient without noticeable effect. Were this patient still presumed to have intractable catatonia, the next step would involve electroconvulsive therapy. However, this more invasive procedure would inflict unnecessary side effects that would be of no benefit in the case of malingering. The treatment would not address her issues of multiple psychiatric illnesses including major depressive disorder and benzodiazepine use disorder, as well as her lack of resources such as food, social supports, and shelter. Instead, we stress that the diagnosis of malingering must be made to shift strategies from medical management to goals around social support services and targeted treatment of other psychiatric issues. This case report adds to the limited existing literature on volitional feigning of catatonia and highlights the need of physicians to accurately distinguish malingering from catatonia, which ultimately leads to better care for the patient.

## Data Availability

The patient data used to support the findings of this study have not been made available because of the need to protect patient privacy.

## References

[1] Fink M., Taylor M. A. (2009). The catatonia Syndrome. *Archives of General Psychiatry*.

[2] Penland H. R., Weder N., Tampi R. R. (2006). The catatonic dilemma expanded. *Annals of General Psychiatry*.

[3] American Psychiatric Association (2013). *Diagnostic and Statistical Manual of Mental Disorders, Fifth Edition (DSM-5)*.

[4] Resnick P. J., Knoll J. (2005). Faking It: How to Detect Malingered Psychosis. *Current Psychiatry*.

[5] Wong J. W., Williams S. R. (2017). The wandering woman: a case study of catatonia vs factitious disorder. *Hawai'i Journal of Medicine & Public Health : a journal of Asia Pacific Medicine & Public Health*.

[6] Wiener M. D., Pauline K. (1990). A case of conversion catatonia misdiagnosed for 24 years. *Jefferson Journal of Psychiatry*.

[7] Hopkins A., Clarke C. (1987). Pretended paralysis requiring artificial ventilation. *British Medical Journal (Clinical Research Ed.)*.

[8] Roi C., Verret L., Peet B., Conrad E. J. (2020). Treatment of a complex case of catatonia and conversion features with electroconvulsive therapy in a 14-year-old male. *Ochsner Journal*.

[9] Harris M. R. (2000). The malingering of psychotic disorders. *Jefferson Journal of Psychiatry*.

